# Editorial: Neuroscience and new music: Assessing behavioral and cerebral aspects of its perception, cognition, induction, and entrainment

**DOI:** 10.3389/fpsyg.2022.979570

**Published:** 2022-07-27

**Authors:** Thomas Lundy, Clara James, Mark Reybrouck

**Affiliations:** ^1^Cuttlefish Arts, Wheat Ridge, CO, United States; ^2^Department of Mathematics, University of Colorado Denver, Denver, CO, United States; ^3^Geneva Musical Minds Lab (GEMMI lab), Geneva School of Health Sciences, University of Applied Sciences and Arts Western Switzerland HES-SO, Geneva, Switzerland; ^4^Faculty of Psychology and Educational Sciences, University of Geneva, Geneva, Switzerland; ^5^Musicology Research Group, Faculty of Arts, KU Leuven-University of Leuven, Leuven, Belgium; ^6^IPEM, Department of Art History, Musicology and Theatre Studies, Ghent, Belgium

**Keywords:** New Music, neuroscience, editorial, cognition, music perception

Defining “New Music” is challenging both temporally (there is no clear starting point) and musically. It comprises any musical idiom diverging from the common practice period, unfamiliar to many listeners. Unfamiliar because of the partial or total abandon of tonality, compositional conventions, and the utilization of unusual sounds and rhythms. All of these aspects require learning new ways of arranging musical notes according to the idiom of each composer or even each piece, soliciting effortful new cognitive and cerebral strategies from the listener. New Music, therefore, is an umbrella term that embraces diverse musical genres: Musique Concrète, electronic synthesis, Elektronische Musik, the controversial second Vienna School, free and serial atonal and pointillistic compositions, spectral music, microtonal music, and many others. One may argue that popular genres such as electronics, dance music, Ambient Music, Sound Art, and Postclassical Minimal Music can join the list. Nowadays, there is a dissipation of the barriers between so-called serious music and more popular music—they all rely on musical sounds and rhythms and their possible transformations as essential constituents.

We launched the Special Research Topic on “*Neuroscience and New Music: Assessing Behavioral and Cerebral Aspects of Its Perception, Cognition, Induction, and Entrainment*” to address the growing need for theoretical and empirical grounding regarding New Music's perception, valuation, and production. Two overarching themes were specified initially: (i) the actual processing of New Music, with a distinction between perception, cognition, induction, and entrainment, and (ii) the neural correlates of these levels of processing and the neuroimaging techniques for their assessment.

The answer to the call for papers was not abundant, which was somewhat unexpected, given the current explosion of music and brain studies. Even if the COVID-19 crisis curtailed and even derailed some exciting proposals, narrowing the scope to New Music reduced the pool of relevant studies, clearly illustrating the salient character of this Research Topic that is underrepresented in the literature.

We finally selected ten papers revolving mainly around topics as divergent as embodied cognition, creative composing, behavioral responses, perceptual issues, aesthetic judgment, predictive processing, the role of technology, and electrophysiological and fMRI measurements.

Foster et al. compared the tempo judgment of disk jockeys (DJ), percussionists, and melodic instrumentalists (all professionals) with untrained controls. The authors show that there is no difference in performance between DJs and other professional musicians in any tempo range, i.e., DJs are on par with professional musicians regarding tempo judgment. The book review by Besada sets Mariusz Kozak's 2020 volume within the broader context of rhythm and musical time. In particular, Besada draws attention to Kozak's emphasis on embodiment and enaction to target the listener's experience while noting that more high-level and abstract representations of time are valuable tools for composers. Besada also commends Kozak for conducting original empirical research to support his reasoning. The contribution by Arkhipova et al. examines the Different Hearing Program (DHP)—which involves creative group composing in the classroom. The authors applied functional brain imaging and behavioral techniques to determine if DHP induces changes in subjective appreciation of different classes of music. The results imply that DHP training altered the activation of functional brain networks, with default mode network activation increasing and executive network activation decreasing in relation to creative thinking. The study by Dauer et al. explored whether inter-subject correlation (ISC) of electroencephalographic responses (EEG) can capture engagement with minimalist music. The authors collected EEG and continuous behavioral (CB) data while 30 adults listened to an excerpt from Steve Reich's *Piano Phase*, three controlled manipulations of Piano Phase, and a popular-music remix of the work. Results show that EEG responses and CB ISC levels were highest for the remix stimuli and lowest for the most recurrent manipulations. Godøy's contribution discusses the concept of the sound object, as embodied in the *Musique Concrète* of Pierre Schaffer. Godøy argues that the sound object is relevant to many musical genres and styles, not just Musique Concrète. Furthermore, he posits that sound objects can better encapsulate time-dependent music features, i.e., various dynamic, textural, timbral, and expressive envelopes, than traditional Western music theory frameworks. Touizrar et al. examines the relationship between the perception of repetition, comprehensibility, and aesthetic judgment in the domain of post-tonal music. Sixty participants identified repetition in 14 three-minute excerpts from ensemble and orchestra pieces selected from three categories: Tonal, Modernist, and Post-1970. Statistical analysis demonstrates a degree of independence between repetition and aesthetic preference. The study by Færøvik et al. notes that the temporal, repetitive nature of musical rhythms is ideal for investigating the phenomena of auditory repetition suppression and omission activation. The study successfully replicated previous findings that left dominant superior temporal brain activation decreases during repetition of an unaltered rhythmic stimulus and right lateralized middle temporal brain activation increases in response to omissions. Generalized activation in error detection areas likely evidenced working memory involvement. These results show that tailored musical stimuli in an fMRI setting permit robust investigation of such neural phenomena. Washburn et al. is a detailed examination of the interaction between acoustic transmission latency (ATL) and asynchrony between performers. The authors found that **n**etworked music performances create longer ATL between performers than traditional in-person settings and, conversely, longer transmission latencies and more significant temporal asynchrony between performers. The perspective article by Mencke et al. notes that the tonal or metrical hierarchy of atonal music exhibits low predictability, whereas Western tonal music is more predictable owing to its hierarchical structure. Thus, listening to atonal music requires generating predictive models. This behavior is characterized in behavioral neuroscience as fulfilling an innate drive to reduce uncertainty but has received little attention in empirical music research. They also suggest new research avenues for deepening our understanding of the aesthetic experience of atonal music in particular and revealing core qualities of the aesthetic experience in general. Finally, Phillips et al. examines listeners' segmentation decisions in a piece of contemporary music, Ligeti's *Fanfares, via* the Practice & Research in Science & Music (PRiSM) Perception smartphone App. Listeners tapped when they felt that a section had ended. Listeners showed high levels of agreement. Moreover, familiarity with contemporary repertoire did not seem to influence the responses, and the differential effect of musical training was small.

In order to grasp New Music, we must adopt alternate tonal and rhythmic frameworks. These include embodiment, attention on recurring or missing elements, innovative thinking, perceiving music as sound objects, and more. Using functional brain imaging (fMRI, EEG) may allow investigating how the brain can process these new musical texts into coherent representations. Clearly, this field requires more comprehensive and well-coordinated research. We hope this Research Topic contributes to that.

## Author contributions

All authors listed have made a substantial, direct, and intellectual contribution to the work and approved it for publication.

## Conflict of interest

Author TL was employed by Cuttlefish Arts. The remaining authors declare that the research was conducted in the absence of any commercial or financial relationships that could be construed as a potential conflict of interest.

## Publisher's note

All claims expressed in this article are solely those of the authors and do not necessarily represent those of their affiliated organizations, or those of the publisher, the editors and the reviewers. Any product that may be evaluated in this article, or claim that may be made by its manufacturer, is not guaranteed or endorsed by the publisher.

